# From leaf and branch into a flower: *Magnolia* tells the story

**DOI:** 10.1186/1999-3110-55-28

**Published:** 2014-03-01

**Authors:** Wen-Zhe Liu, Khidir Hilu, Ya-Ling Wang

**Affiliations:** 1 Key Laboratory of Resource Biology and Biotechnology in Western China (Ministry of Education), School of Life Sciences, Northwest University, Xi’an, China; 2 Department of Biological Sciences, Virginia Tech, Blacksburg, VA 24061 USA; 3 Xi’an Botanical Garden, Xi’an, China

**Keywords:** Angiosperm, Carpel, Origin, Comparative anatomy, Flower, *Magnolia*

## Abstract

**Background:**

In the classical doctrines, *Magnolia* was frequently considered the archetype among flowering plants, and its conduplicate carpel with marginal placentation was assumed to be derived from a leaf-like organ bearing ovules along its margins. Although the robustness of this concept has been seriously questioned by advances in botanical research, especially the emergence of *Magnolia* deeper in the angiosperm tree of life in molecular systematics, it remains the most-taught interpretation for the origin of carpels.

**Results:**

To test the validity of this classical doctrine, we performed comparative anatomical analyses of the vascular bundles in the flowers of *Magnolia* using fine (8-μm) paraffin -sections. We document the presence of two independent vascular systems in the carpels: the collateral bundles of the dorsal and ventral veins arising from the stelar bundle, and the amphicribral ovular bundles arising from the cortical bundles. This observation in conjunction with data from other fields concurrently suggests that the ovary wall is equivalent to a foliar organ whereas the placenta represents an ovule-bearing shoot.

**Conclusions:**

Our observation on the former model plant, *Magnolia*, nullifies the classical doctrine of carpel evolution and supports the Unifying Theory. This conclusion prompts a reconsideration of the concept of angiosperm flower evolution.

**Electronic supplementary material:**

The online version of this article (doi:10.1186/1999-3110-55-28) contains supplementary material, which is available to authorized users.

## Background

Before the debut of molecular phylogenetics, angiosperm systematics were dominated by a so-called classical botanical doctrine, according to which *Magnolia* was one of the most primitive angiosperms and its conduplicate carpel with marginal placentation was taken as the most primitive condition among angiosperms (Eames 1931; Thomas 1931; Bailey and Nast 1943; Bailey and Swamy 1951; Eames 1961; Fahn 1982; Ueda 1986; Thorne 1996; Xu and Rudall 2006). Eames (1931) once stated that the primitiveness of this kind of carpel is unequivocal. Detailed studies on the gynoecia in the Magnoliaceae have demonstrated that this oversimplification cannot be corroborated (Tucker 1961; Eyde 1975; Tucker 1975; Ueda 1982; Endress et al. 2000; Xu and Rudall 2006; Fu et al. 2009; Deroin 2010). Furthermore, the traditional phylogenetic position of the Magnoliaceae as sister to remaining angiosperms (Cronquist Magnolialean hypothesis; Cronquist 1988;) is no longer tenable and has been replaced by the APG system (Qiu et al. 1999; Hilu et al. 2003; APG 2009; Chase and Reveal 2009; Vialette-Guiraud and Scutt 2009; Soltis et al. 2011). However, the structure of the carpel in the first diverging angiosperm family Amborellaceae in the current angiosperm phylogeny remains unknown, and thus we lack a more plausible interpretation for the origins of carpel (Endress and Igersheim 2000; Doyle 2012). As such, the classical doctrine based on *Magnolia* remains to be the most-taught hypothesis for the origin of carpels in classrooms (Eames 1931;Bailey and Nast 1943; Bailey and Swamy 1951; Canright 1960; Eames 1961; Takhtajan 1969; Cronquist 1988). This doctrine may well have misguided the palaeobotanical search for angiosperm ancestors and influenced interpretations of early fossil angiosperms, including *Archaefructus* (Wang and Zheng 2012). Therefore, this traditional, *Magnolia*-based concept of angiosperm carpel evolution persists in the literature, and thus its validity needs to be addressed.

The evo-devo studies indicate ovule/placenta and ovary wall are controlled by two distinct, exclusive sets of genes (Angenent et al. 1995; Rounsley et al. 1995; Pinyopich et al. 2003; Skinner et al. 2004; Yamaguchi et al. 2004; Dreni et al. 2007; Yoo et al. 2010; Li et al. 2011; Mathews and Kramer 2012), suggestive of separate provenances for these two parts. This conclusion is compatible with the Unifying Theory Wang (2010) based on the study of a Jurassic fossil plant with free central placentation Wang (2010) plus morphological, anatomic, and developmental genetic evidence, namely, that the carpel in the classic sense is a composite organ comprising an ovule-bearing shoot and foliar parts enclosing the shoot Wang (2010). A logical inference from this theory is that placenta in angiosperms should have amphicribral vascular bundle, just like that in a young branch. However, this inference still needs to be tested with comparative anatomy. Here our anatomical study on the model plant of the former doctrine, *Magnolia*, provides the first support, to our knowledge, for the hypothesis using empirical data.

## Methods

**Figure 1:**
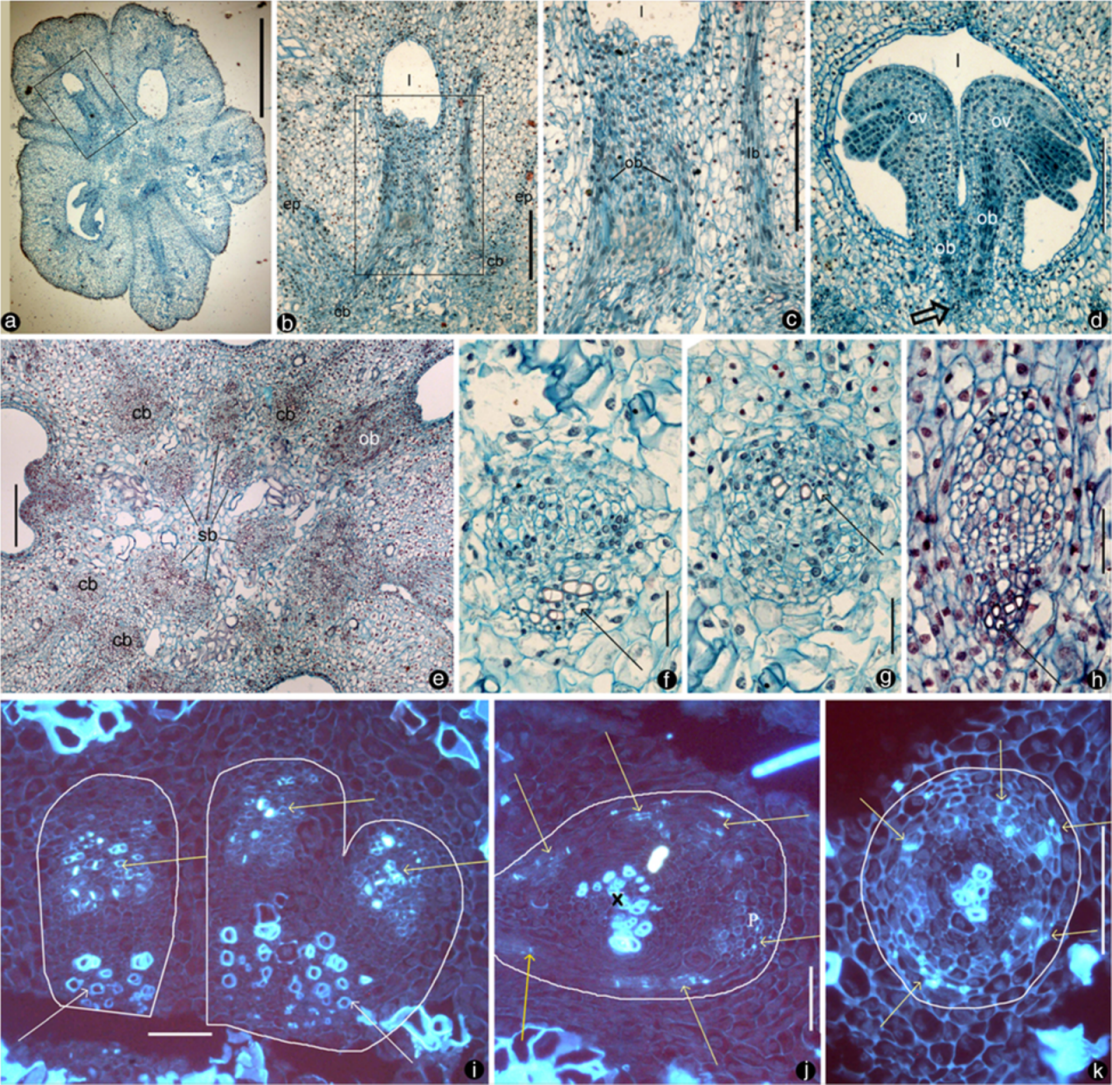
**Cross sections of the gynoecium in** ***Magnolia denudata*** **flower.** *cb*: cortical bundle, *db*: dorsal bundle, *ep*: epidermis, *l*:ovarian locule, *lb*: lateral bundle, *ob*: ovular bundle, *ov*: ovule, *sb*: stelar bundle, *vb*: ventral bundle. Adaxial side is to the bottom. Specimen number NWU00032054-4403, deposited at Northwest University, China. **a**. Cross section of a flower, showing several carpels around the floral axis. Bar = 1 mm. **b**. Detailed view of the rectangle in Figure. 2a, showing cortical bundles giving rise to two ovular bundles as well as a lateral bundle. Bar = 0.2 mm. **c**. Detailed view of the rectangle in Figure 1b, showing two ovular bundles and a lateral bundle. Bar = 0.2 mm. **d**. Two ovules in the same ovary arising from the same ovular bundle (arrow). Bar = 0.2 mm. **e**. Floral axis with stelar bundles, cortical bundles, and their relationship with carpel boundaries and ovular bundles. Bar = 0.2 mm. **f**. Collateral stelar bundle with adaxial xylem (arrow). Bar = 50 μm. **g**. Amphicribral cortical bundle with close-to-center xylem (arrow). Bar = 50 μm.**h**. Collateral dorsal bundle with adaxial xylem (arrow). Bar = 50 μm. **i**. Three collateral stelar bundles with adaxial xylem (white arrows) and abaxial phloem (yellow arrows). Bar = 50 μm. **j**. Amphicribral ovular bundle with phloem (P) (yellow arrows) surrounding the xylem (X) in the center (circle). Bar = 50 μm. **k**. Amphicribral cortical bundle with phloem (yellow arrows) surrounding the xylem in the center (white). Bar = 0.1 mm.

**Figure 2:**
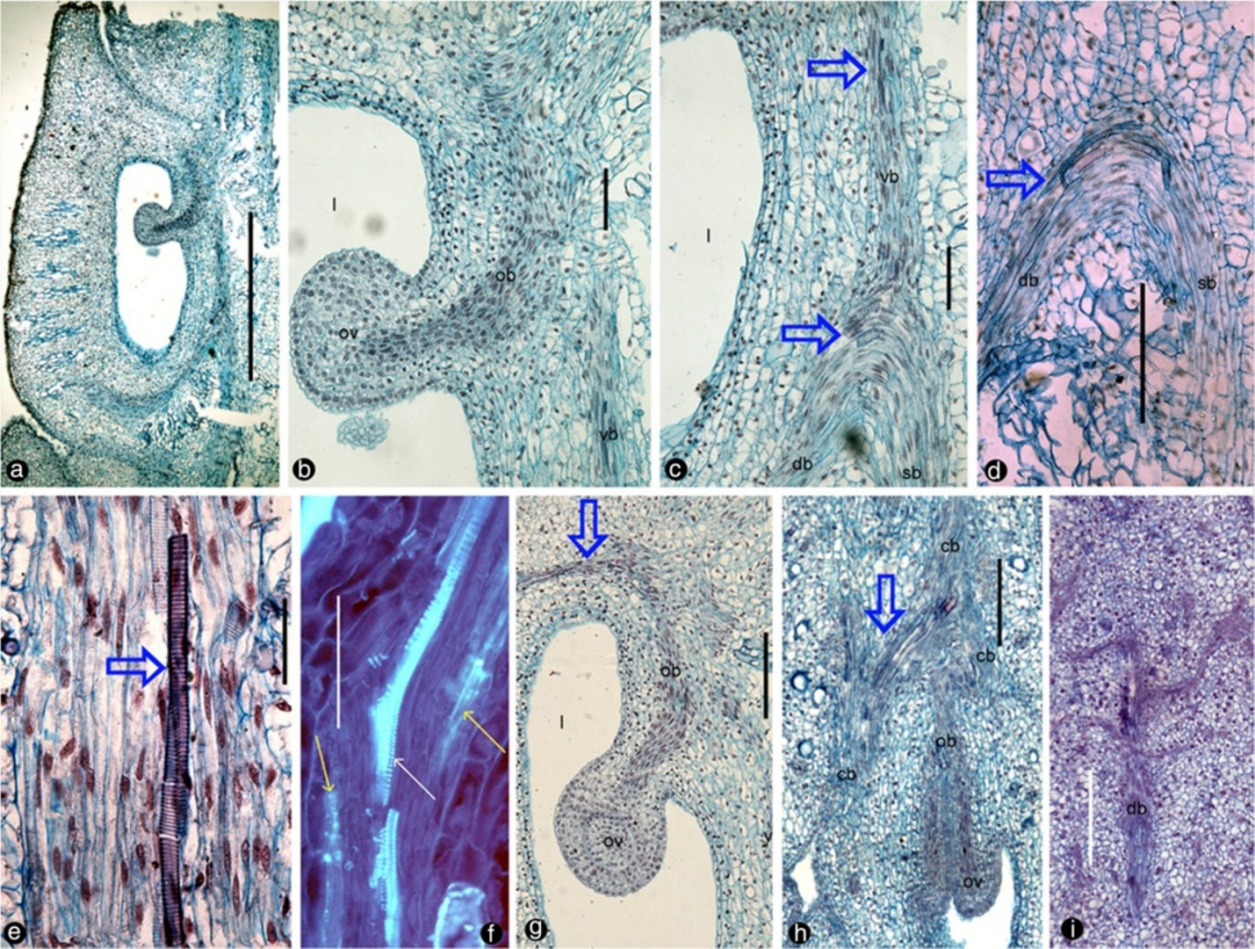
**Longitudinal sections of** ***Magnolia denudata*** **flower.** For abbreviations, see caption for Figure 1. Specimen number NWU00032054-4405, deposited at Northwest University, China. **a**. Radial section showing a carpel and its relationship to the floral axis to the right. Bar = 1 mm. **b**. Detailed view of the ovule in Figure 2a. Note the relationship between the ovule, descending ovular bundle, and ventral bundle. Bar = 0.1 mm. **c**. Detailed view of the dorsal bundle in Figure 1a. Note the relationship among the ascending ventral bundle (vb, blue arrows), descending dorsal bundle (db, blue arrows), and ascending stelar bundle (sb). Bar = 0.1 mm. **d**. Dorsal (db, blue arrows) and stelar bundles (sb) in another carpel. Bar = 0.2 mm. **e**. Vessel element (blue arrow) in an amphicribral cortical bundle, with central xylem. Bar = 50 μm. **f**. Xylem (tracheids, white arrow) sandwiched between phloem (sieve elements, yellow arrows) in the amphicribral ovular bundle. Bar = 0.1 mm. **g**. An ovular bundle (ob) supplying the ovule and ascending along the carpel margin (blue arrow). Bar = 0.2 mm. **h**. Tangential section showing anastomosing cortical bundles (cb) connected to ovular bundles (ob). Note the branching (blue arrow) of the cortical bundles, and their relationship to the ovular bundles. Bar = 0.2 mm. **i**. Tangential section showing a dorsal bundle branching pinnately. Bar = 0.2 mm.

Flower buds and fructifications at various developmental stages were collected during March 2011 and 2012 from trees of *Magnolia denudata* [NWU00032054] cultivated on the campus of the Northwest University, Xi’an, Shaanxi, China (We are permitted to collect flower of *M. denudata* for scientific research by Xi’an municipal greening committee). *Magnolia denudata* was chosen because of its accessibility as a cultivated tree on the campus of Northwest University. Since this is a hexaploid species with 2n = 6× = 114, we expanded the scope of our study to avoid potential influence of ploidy on our results. A diploid species *Magnolia championii* (2n = 2× = 38) was also examined. The materials from both were fixed with FAA and then used in the preparation of 8-μm thick paraffin sections following the routine methods (Ruzin 1999). Parts of the paraffin sections were stained with Safranin O and Fast Green, critically examined and photographed using a Nikon Eclipse 50i microscope with a Nikon DS-Fil digital camera (Figures 1a-h, 2a-e, g-i). The other paraffin sections were stained with Aniline Blue, examined and photographed after excitation at 365 nm using a Leica DML epifluorescence microscope with a Leica DC300F camera (Figures 1i-k, 2f). The figures are organized for publication using Adobe Photoshop 7.0. All sections are deposited at Northwest University, Xi’an, Shaanxi, China.

The anatomy and morphology of *Magnolia* flowers were based on the observation and careful tracing of vascular bundles within 25 flowers from 5 trees. The organizations of vascular bundles were consistent without exception.

We will apply two frequently used terms that require clarification: 1) amphicribral bundle, which designates a vascular bundle that has its xylem surrounded by the phloem (as a protostele in early land plants) and 2) collateral bundle, which defines a vascular bundle that has adaxial xylem and abaxial phloem (as a vein in a typical macrophyll). (For more information, see Ye 2002).

## Results

**Figure 3:**
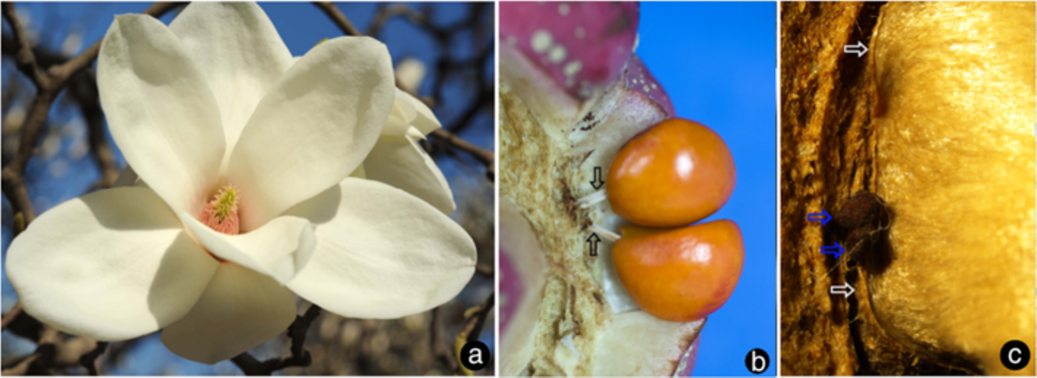
**Flower and fruits of** ***Magnolia denudata*** **.** **a**. A blooming flower. **b**. Red seeds hanging on the placenta after the ovary wall is shed. Note the connections (arrows) between the placenta and seeds. **c**. Two aborted ovules (blue arrows) independent of their ovary wall in the background. Note the border of the ovary wall (white arrow).

**Figure 4:**
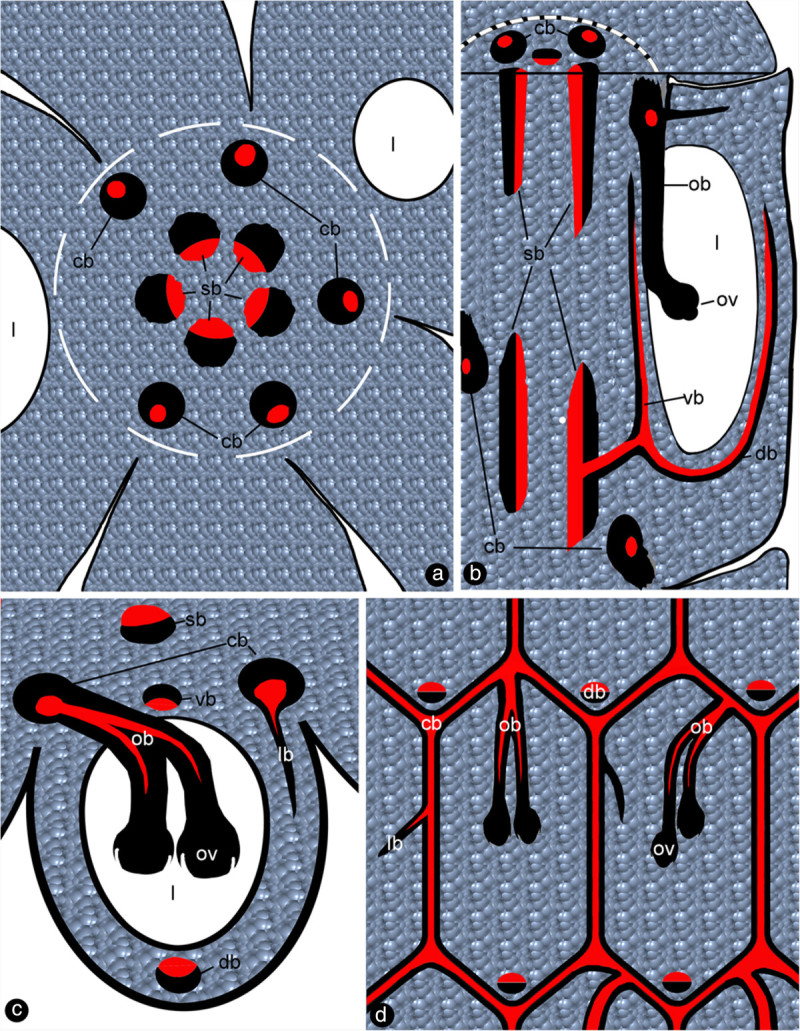
**Sketches showing the vascular anatomy of** ***Magnolia denudata.*** For abbreviations, see caption for Figure 1. Red color for xylem, and black for phloem. **a**. Cross section showing ovarian locules, carpels around the floral axis (white broken line), collateral stelar bundles in the center, and amphicribral cortical bundles in the cortex. **b**. Three dimensional diagram showing a carpel attached to the floral axis, collateral stelar and amphicribral cortical bundles, ventral and dorsal bundles derived from the stelar bundles. **c**. Cross section of a carpel showing the deployment of collateral stelar bundle, collateral ventral and dorsal bundles, amphicribral cortical and ovular bundles, and lateral bundle. **d**. Anastomosis of cortical bundles and their relationship with ovules and lateral bundles in carpels.

**Figure 5:**
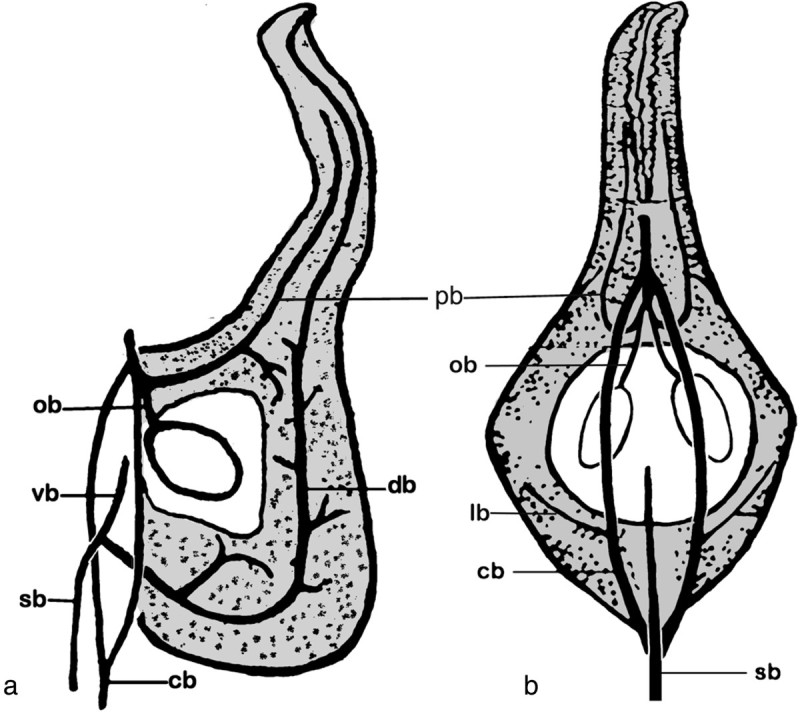
**Diagrams of vascular patterns of carpels in** ***Magnolia denudata*** **(redrawn and modified from Canright,** 1960**).** *cb*: cortical bundle, *db*: dorsal bundle, *lb*: lateral bundle, *ov:* ovule, *ob*: ovular bundle, *pb*: placenta bundle, *sb*: stelar bundle, *vb*: ventral bundle. **a.** The lateral view. **b.** The ventral view, look into the flower periphery.

The ovary wall is shed from the mature fruit while the seeds or aborted ovules still hang on to the placenta/flower axis (Figure 3b). The ovules/seeds are attached to the placenta and are independent of the ovary wall (Figure 3c). The sections of the pre-authentic flowers of *Magnolia denudata* (Figure 3a) demonstrated that the vascular system in the female part of floral axis was composed of two related sets of systems: a stelar system and a cortical system (Figures 1a, e, 4a, 5a, b). The longitudinal sections revealed that the cortical system was derived from the stelar system at the base of the female part of the flower, and became independent of it from there up (Figure 1a-e, etc.). The cortical system comprises of four to six bundles alternating with the stelar bundles and opposing the boundaries between two adjacent carpels in cross section (Figures 1a, e, 4a, 5a, b). In the center of the floral axis, an eustele of four to six anatomosing collateral bundles separated by ground tissue constitutes the stelar system (Figures 1a, e, 4a, 5a, b). The xylem of each collateral bundle faces the center of the floral axis (Figures 1e, 4a). A bundle diverges from a stelar bundle, giving rise to dorsal and ventral bundles in a carpel (Figures 2a, c, 4b, 5a, b). The dorsal bundle descends first and then ascends to the carpel tip (Figures 2a, c, d, 4b), and may branch pinnately (Figure 2i). The ventral bundle may be present or absent (Figure 2c, d), If present, it rises and maintains its isolation from the ovules (Figures 2a-c, g, 4b). Both of the dorsal and ventral bundles are collateral (Figures 1h, 4b, 5a, b). The cortical system anastomoses in the cortex of the floral axis (Figures 1a, e, 4a, b). These cortical bundles are amphicribral, namely, the xylem is surrounded by the phloem (Figures 1g, k, 2e, h, 4a-d). The vascular bundles supplying the ovules are amphicribral (Figures 1j, 2f, 4b-d), almost vertically descending from a cortical bundle to the ovules in the ovary (Figures 2a, b, g, h, 4b, 5a, b). Occasionally lateral bundles of ovary wall are derived from a cortical bundle (Figures 1a-c, 4c, d, 5a, b).

**Figure 6:**
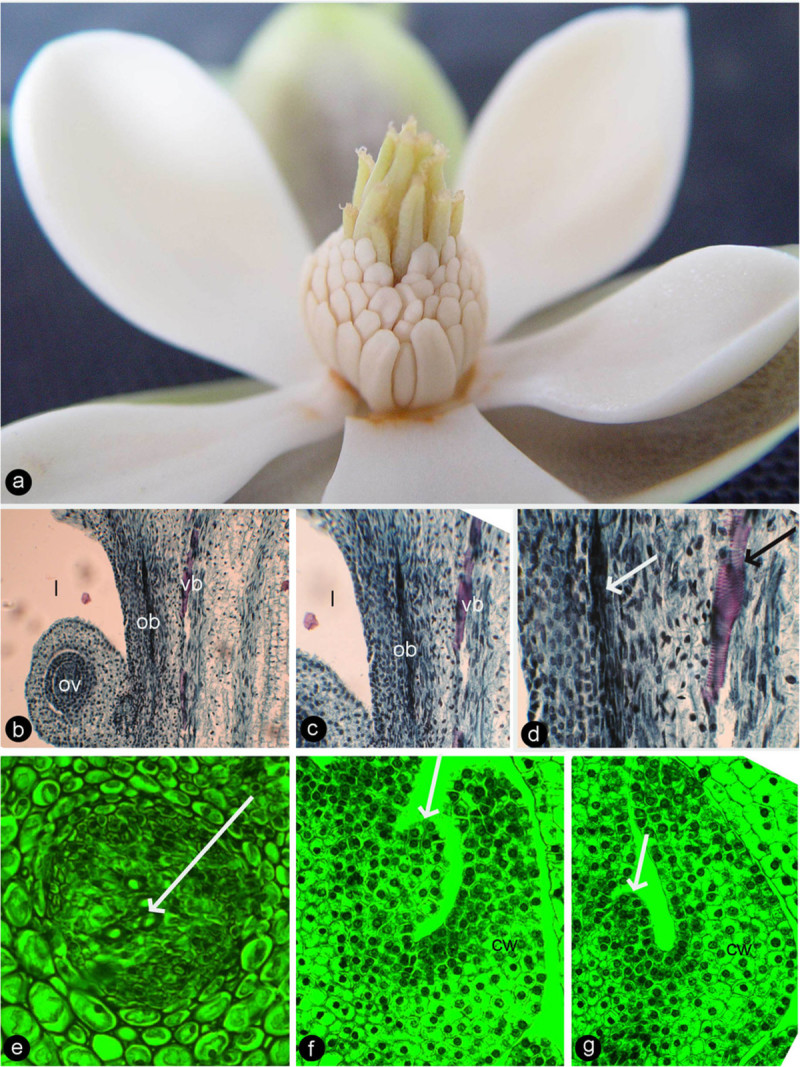
**Flower and its anatomy of** ***Magnolia championii.*** *cb*: cortical bundle, *l*:ovarian locule, *lb*: lateral bundle, *ob*: ovular bundle, *ov*: ovule, *vb*: ventral bundle. **a**. Blooming flower. **b**. Longitudinal sections of a flower, showing relationship between ovarian locule, ovule, ovular bundle, and ventral bundle. **c**. Detailed view of Figure 6b, showing ovular and ventral bundles. **d**. The central xylem (white arrow) in ovular bundle and abaxial xylem (black arrow) in ventral bundle. **e**. Cross section of an amphicribral ovular bundle with central xylem (white arrow) in the placenta. **f**-**g**. Longitudinal sections of carpels in their early development showing carpel wall (CW) and placenta (arrows).

The flowers of *Magnolia championii* (Figure 6a) are similar in anatomy and development to those of *M. denudata* (Figure 3a). In early development, the young ovule appears attached to the axis and subtended by the developing ovary wall (Figures. 6f–g). The enclosure is completed later in the development when the carpels meet the floral axis from the sides and the top. The ventral bundles, if present, ascend from the bottom and remain isolated from those supplying the ovules, which descend from the top (Figures 6b, c). The xylem is positioned in the center of the bundles supplying the ovules while positioned abaxially in the ventral bundle (Figure 6d). The central position of xylem becomes especially conspicuous in the bundle supplying the ovules/seeds in more mature materials (Figure 6e).

## Discussion

The existence of vascular conservatism has been a highly debated topic (Eames 1931; Puri 1951; Carlquist 1969; Schmid 1972). Actually none of plant features, including DNA sequences, morphology, anatomy, and ontogeny, should be designated as the only criterion used to evaluate any specific botanical question. Neither, any one source of evidence should be over-valued or downgraded, and conclusion based on one type of data should be tested and confirmed by data from other disciplines. The following discussion and conclusion are based on 1) that all the criticisms raised against vascular conservatism by Carlquist (1969) are not applicable on our case because Carlquist even did not mention phloem or amphicribral bundles throughout the paper, 2) that our anatomy- and development-based conclusion is supported by other independent research (see below). Therefore, we hereafter assume the constant distinction between collateral and amphicribral bundles to be meaningful for the following homological analysis.

Although it is in disagreement with various studies (Canright 1960; Tucker and Gifford 1966; Eyde 1975; Tucker 1975), the most classic carpel theory is still widely taught in botanical class (Canright 1960; Eames 1961; Takhtajan 1969; Cronquist 1988; Thorne 1996). The current concept of angiosperm phylogeny places *Amborella* as sister to remaining angiosperms (Qiu et al. 1999; APG 2003; Hilu et al. 2003; APG 2009; Soltis et al. 2011), but an alternative theory for flower evolution based on morphological/anatomical investigations for this genus that can completely substitute the classical one is not available. Although ascidiate carpel is inferred ancestral in angiosperms (Endress and Doyle 2009), it cannot be taken as a shared feature for ANITA if the Illiciaceae are taken into consideration (Endress and Igersheim 2000). The Unifying Theory, a hypothesis founded on a Jurassic plant with free central placentation, dissects a carpel into an ovule-bearing shoot (placenta) and its enclosing foliar structure (Wang 2010). This treatment was suggested previously by the Gonophyll Theory (Melville 1962) and is compatible using data from various fields of botany, including development, morphology, anatomy, gene function analysis, and systematic analysis (Herr 1995; Rounsley et al. 1995; Hickey and Taylor 1996; Skinner et al. 2004; Dreni et al. 2007; Doyle 2008; Zheng et al. 2010; Carlsbecker et al. 2013). If this treatment is correct, a logical inference is that there should be vascular bundles of radial symmetry (namely, amphicribral bundles) in the placenta. We attempt to empirically test for the first time the above inference using the anatomical, morphological, and developmental features. Whether *Magnolia*, the assumed model plant in the traditional doctrine, favors this theory becomes very critical and important for angiosperm systematics.

The significance of recognizing amphicribral bundle in placenta is greatly enhanced if the fossil record and outgroup information is taken into consideration. Axillary branch is shared by most seed plants except Cycadales (Crane 1985), and a typical branch trace is formed by fusing two facing collateral bundles (Figure one hundred point two of Fahn 1982). The ovules in all (at least extant) gymnosperms are borne on shoots rather than leaves (Florin 1949, 1951; Rothwell and Mapes 2001; Zhou et al. 2007; Wang et al. 2012; Rothwell and Stockey 2013), although the term megasporophyll is frequently used. The amphicribral/concentric vascular bundles in these shoots supply the ovules, in full agreement with the fossil record. Early fossil seeds are usually supplied by a central terete xylem surrounded by delicate cells or cavity (decayed phloem?) [*Genomosperma kidstoni* in Plate I, Figures eight (Long 41960a); *Lyrasperma scotica* in Plate II, Figure twenty three (Long 1960b); *Dolichosperma sexangulatum* in Plate IV, Figure fifty six (Long 1961); *Elkinsia polymorpha* in Figure fourty four (Rothwell et al. 1989); *Ruxtonia minuta* in Plate 3, Figure 2 and Text-Figure 5f (Galtier et al. 2007); *Cardiocarpus samaratus* in Figure twelve c (Wang et al. 2003)] and they are terminal on shoots. Similar configuration has also been seen in the placental bundle in an anatomically preserved angiosperm, *Beardia* (Juglandaceae), from the Eocene of Canada (igures five, seven, eight of Elliott et al. F2006). Since all ovules in these seed plants are borne on shoots, it is rather expected that this rule is also applicable for angiosperms, a subset of seed plants. This hypothesis is strongly favored by *Xingxueanthus*, a fossil reproductive organ with all ovules attached to a central column within the ovary Wang and Wang, 2010). The above conclusion based on morphology is confirmed by the study on genetically manipulated *Arabidopsis*, in which ovules are borne on placenta without any coverage of carpel wall (Roe et al. 1997). Finally, amphicribral bundles appear to be regularly present in the placentae of various angiosperms (Lersten and Don 1966; Tucker 1975; Dave et al. 1981; Guo et al. 2013; Lersten and Don 1966; Kapoor 1973, 1995; Nuraliev et al. 2011; Schmid 1978; Tucker 1975; Von Balthazar and Endress 2002; Wang and Pan 1998). Taken all together, the placenta in angiosperm is most logically derived from an ovule-bearing shoot with an amphicribral bundle.

As recognized previously, there are cortical and central vascular systems in female section of the Magnoliaceae flower (Canright 1960; Tucker 1961; Ueda 1986; Deroin 1999). Tucker (1961) partially discrediting Canright’s interpretation, noticed the existence of two vascular systems and different (“concentric” and collateral) vascular bundles in *Michelia champaca*. Our observation casts further doubt over Canright’s generalization. For example, our observation indicates that dorsal and ventral bundles of *Magnolia* carpels are connected with the central stelar system, while the placental bundles are connected with the peripheral cortical system. The dorsal and ventral bundles in the carpel of *M. denudata* are collateral with the orientation just as assumed in the classic doctrine (Figures 1h, 2c, d, 4a, b). However, unlike the classic doctrine assumption, the ventral bundle is not associated with the ovules and the ovary wall/carpel is isolated from the placenta. This independence of ovules from the ovary wall is obvious as seeds are still hanging from the floral axis/placenta after the ovary wall falls off (Figure 3b, c). These results are in conflict with the widely accepted carpel theory (Bailey and Swamy 1951; Eames 1961), which appeared favored by the study of floral anatomy of Magnoliaceae (Canright 1960). However, other studies are more or less compatible with ours. The vascular bundles supplying the ovules are amphicribral and derived from cortical amphicribral bundles (Figures 1a, d, g, j, 2e-g, 4b-d, 6e), as noted previously (Ueda 1986). The cortical position of the ovule-supplying bundles in floral axis may have resulted from the absorption of ovule-bearing shoot into the flower axis cortex, just as decurrent leaf bases fuse into stem cortex (Kaplan 2001). The deployment of xylem and phloem in these vascular bundles is similar to that seen in a typical axis and protostele (as in *Rhynia*), implying an axial nature for the placental bundles. This situation is in agreement with Hickey and Taylor (Hickey and Taylor 1996) and Herr (1995). Herr (1995) states that “universally throughout vascular plants without exception, sporangia [including ovules, annotated by the present authors] are not literally borne on leaves”. Such axial bundles would be apparently out of place in a carpel if the latter was assumed to be foliar in nature and had only collateral bundles (Arber 1950). At least some of the assumed “inverted” ventral ovule-bearing bundles in carpels appear more like xylem surrounded by phloem (Figure two hundred twenty five point two in Fahn 1982). In fact, this inconsistency between data and interpretations is not restricted to *Magnolia*. Amphicribral bundles have been shown to be related to ovules/placenta in various angiosperms (Papaveraceae (Kapoor 1973;, 1995); Leguminosae in Figure 3 (Lersten and Don 1966); Gesneriaceae in Figures eleven to thirteen (Wang and Pan 1998); Buxaceae in Figures ten Q and eghty two N (Von Balthazar and Endress 2002); Actinidiaceae (Guo et al. 2013)). These families span the angiosperm tree of life from the magnoliids to the monocot and terminal eudicot lineages. Most interestingly, a recent study on the fruits of Hydatellaceae (Nymphaeales), a member of the second diverging angiosperm lineage (Saarela et al. 2007; APG 2009), demonstrates that the xylem is positioned in the center of the placental bundle (Figure 6, d in Sokoloff et al. 2013). This frequently ignored inconsistency between the classic doctrine and observation in its model plant *Magnolia* as well as other plants cast serious doubt on the classic doctrine. Considering the serious doubt based on studies of so many plants, it is imperative to discern whether *Amborella*, the first diverging angiosperm lineage, possesses a similar amphicribral ovular bundle to shed further light on angiosperm evolution and systematics. If future studies confirmed this, it would suggest that carpels of all angiosperms are derived following the same Bau-plan.

The problem of the persistence of the traditional doctrine was compounded by various factors. Previous documentations of *Magnolia* may have been misleading, or misled, due to the pre-existing conception. For example, a relatively uniform vascular pattern was given for most genera in Magnoliaceae, in which all ovules are related to the ventral veins (.) (Canright *a. st. b*^1960^; Deroin Figure eight; Deroin 1999). In fact, the ovular bundle (*ov. b*.) in *Magnolia* is apparently connected with one of the cortical bundles (*cort. b*.) rather than an ascending stelar bundle (*a. st. b*.) . However, the ovular bundle illustrated appeared to be connected to the ascending stelar bundles in Figure seventeen of the same paper (Canright 1960). This inconsistency between data and interpretation in the same paper implies that the conclusion put forth in the paper is dubious and requires a close re-examination. It is of importance to note that Figure eight in Canright’s paper is in agreement with our observations and is a direct photograph rather than a hand-drawn illustration, thus is more reliable. Probably it is the clearing technique that prevented Canright from interpreting the vascularization correctly (Deroin 2010). Unfortunately, Canright’s interpretation became widely accepted and rarely, if ever, examined. For example, Deroin (1999) apparently adopted Canright’s interpretation uncritically and thus incurs doubt over his own conclusions. Our results, together with Canright’s mismatch of vascular bundles, demand a serious reconsideration and correction on thinking about floral vasculature in Magnoliaceae.

Since a previous study (Ueda 1986) indicates that there is a basic plan of floral vascular system in the Magnoliaceae consistent with our finding, we assume our new observation and conclusion may be applicable throughout the whole family. One might argue that the *Magnolia* case should not be generalized considering the great diversity of flowers and carpels in angiosperms. However, so far there are only few studies focusing on amphicribral bundles in flower/fruit (Kapoor 1973;, 1995). We hypothesize that amphicribral bundle is a regular presence in angiosperms’ placenta. We hope that more extensive studies, especially that on early diverging angiosperm lineages, in the future will test the validity of this hypothesis.

Considering the sister relationship between angiosperms and gymnosperms, correlating carpels in angiosperms with their counterpart structures in gymnosperms has been a lasting challenge. Melville (1962) advanced the Gonophyll Theory, suggesting that the cortical vascular bundles are remnants of vasculature of the axillary shoot. Ueda and others (Ueda 1986) rejected this theory based on the uniform plan in all appendages in Magnoliaceae and non-axillary nature of cortical vascular system. Although he noted that the cortical strands are restricted above the region without axillary buds while the axillary buds are only seen in region without cortical bundles (Ueda 1986), he did not explore the implication of this mutual exclusive distribution of cortical bundles and axillary buds. It is very likely that the disappeared axillary buds may have been transformed into cortical vascular bundles supplying ovules. It has been known for quite a while that there are two growth domains in a carpel corresponding to placenta and ovary wall (Taylor 1991; Doyle 1994; Rounsley et al. 1995; Skinner et al. 2004; Doyle 2008; Wang 2010). Studies of gene expression patterns in flowers of model plants including *Arabidopsis*, *Petunia*, and *Oryza* also indicate that STK, FBP7, FBP11, AGL11 and OsMADS13 are restricted to placenta/ovules (Angenent et al. 1995; Rounsley et al. 1995; Pinyopich et al. 2003; Skinner et al. 2004; Dreni et al. 2007; Yoo et al. 2010; Li et al. 2011), while DL, CRC and YABBY are found only in the ovary wall (Skinner et al. 2004; Yamaguchi et al. 2004; Dreni et al. 2007; Li et al. 2011). This implied that the placenta was a distinct floral organ equivalent to a secondary shoot independent of the carpel and was recruited onto the ovary wall later in angiosperms (Angenent et al. 1995; Roe et al. 1997; Skinner et al. 2004). This conclusion is plausible considering that ovules are borne on fertile shoots in some gymnosperms (Florin 1949, 1951; Eames 1952; Zhou and Zheng 2003; Zheng and Zhou 2004; Zhou et al. 2007; Wang 2010; Rothwell and Stockey 2013), and that ovule formation has nothing to do with carpels in some mutant angiosperms (Scott 1906; Rehder 1911; Angenent et al. 1995; Roe et al. 1997) and gymnosperms (Bierhorst 1971; Biswas and Johri 1997). Our conclusion is also in agreement with the latest progress in botany (Guo et al. 2013).

Challenges against traditional doctrine come from various studies, but these challenges are usually downplayed. Endress (2005) has documented the ovules attached to the dorsal bundle in *Brasenia*. An ovule inserted on a dorsal bundle is apparently unexpected for the assumed primitive conduplicate carpels. The observation of Endress (2005) is apparently in conflict with the traditional doctrine, but Endress left it alone. On page 211 of the same paper, he stated that “all vascular bundles are collateral” in *Brasenia* carpels. However, his statement fully compatible with the traditional doctrine is nullified by his own Figure 2q, r, t, in which the xylem is centrally positioned in the placental bundle. Nuraliev et al. (2011) find ventral bundles isolated from bundles of other lateral flower appendages and mismatch between ventral bundles and carpels in certain species of *Schefflera*. The isolated ventral bundles in *Schefflera*, in contrast to the bundles of other flower parts that may fuse or connect each other, implies the placenta is distinct from other flower part, which are frequently taken as leaf equivalents. The number of ventral bundles is fewer than the number of carpels in certain species of *Schefflera*. If conduplicate or ascidiate carpel were taken as ancestral states in angiosperms according to either the traditional doctrine or APG proposal, there should be a strict one-to-two or one-to-one correspondence between carpels and ventral bundles. Apparently the situation in *Schefflera* does not meet this expectation, casting doubt over both interpretations. It is interesting that the above inconsistencies will disappear when our interpretation is adopted: a placenta is an ovule-bearing branch with an amphicribral bundle that may branch freely and fuse with any parts of carpel wall, including ventral, dorsal veins, or somewhere in between. But it should be kept in mind that there are also exceptions to this rule. For example, an amphicribral bundle may become or appear collateral to its extremity or in highly derived taxa (For example, Araliaceae of Apiales) (Nuraliev et al. 2011). These exceptions do not constitute strong cases against this rule but rather reflect the adaptation of vascular bundles to their function or evolutionary trend. The final goal of science is to find a pattern behind complicated ephemeral phenomena and to make nature predictable. We are happy to see that there are increasing evidence showing that the placental bundles are either amphicribral or derived from such bundles (Dave et al. 1981; Guo et al. 2013; Lersten and Don 1966; Kapoor 1973, 1995; Nuraliev et al. 2011; Schmid 1978; Tucker 1975; Von Balthazar and Endress 2002; Wang and Pan 1998).

Although only one gene mutation (*phan*) is required for a leaf to transform into cylindrical, abaxialized structure with amphicribral bundles (see Figure 2f, 4a-d of Waites and Hudson 1995; Scarpella and Meijer 2004), this mutation does not disturb the close correlations between leaf-like dorsiventral organization and collateral bundles as well as between stem-like radial organization and amphicribral bundles. Therefore, we do not think that our conclusion is in conflict with molecular genetics.

## Conclusions

Our comparative anatomical evidence of Magnoliaceae supports the following conclusion. First, the separation between the ventral + dorsal bundles and cortical + ovular bundles. Second, the amphicribral cortical and ovular bundles imply an axial nature for the placenta. This is compatible with the fact that the precursors of ovules (megasporangia) were originally borne along the terminals of axes, as suggested by studies of a Jurassic angiosperm, *Xingxueanthus*, and other fossil and extant plants (Florin 1949; Florin 1951; Eames 1952; Herr 1995; Zhou and Zheng 2003; Zheng and Zhou 2004; Zhou et al. 2007; Wang and Wang 2010; Carlsbecker et al. 2013; Rothwell and Stockey 2013). This interpretation would render the search for angiosperm ancestors in gymnosperms much less challenging than previously thought, and we anticipate to find some fossil with their ovules in the axils of bracts in the future.

## Authors’ original submitted files for images

Below are the links to the authors’ original submitted files for images. Authors’ original file for figure 1 Authors’ original file for figure 2 Authors’ original file for figure 3 Authors’ original file for figure 4 Authors’ original file for figure 5 Authors’ original file for figure 6
