# Supplementary figures and images for: From leaf and branch into a flower: Magnolia tells the story
Source: Bot Stud. 2014 Mar 1;55:28. doi: 10.1186/1999-3110-55-28 (PMC5432820; doi:10.1186/1999-3110-55-28)

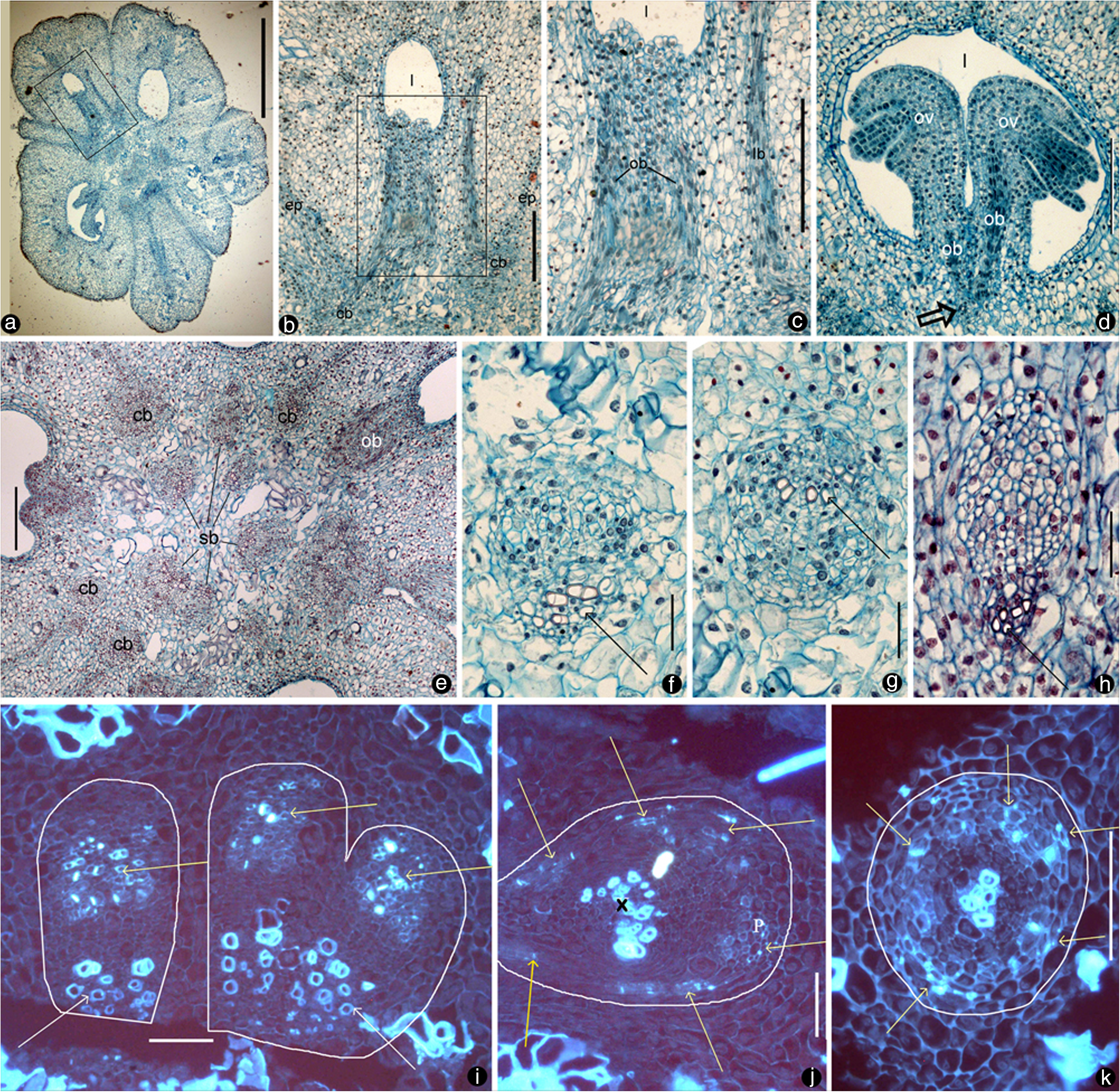

Supplement: Supplementary file 1 — Authors’ original file for figure 1 [file 40529_2014_84_MOESM1_ESM.tif]

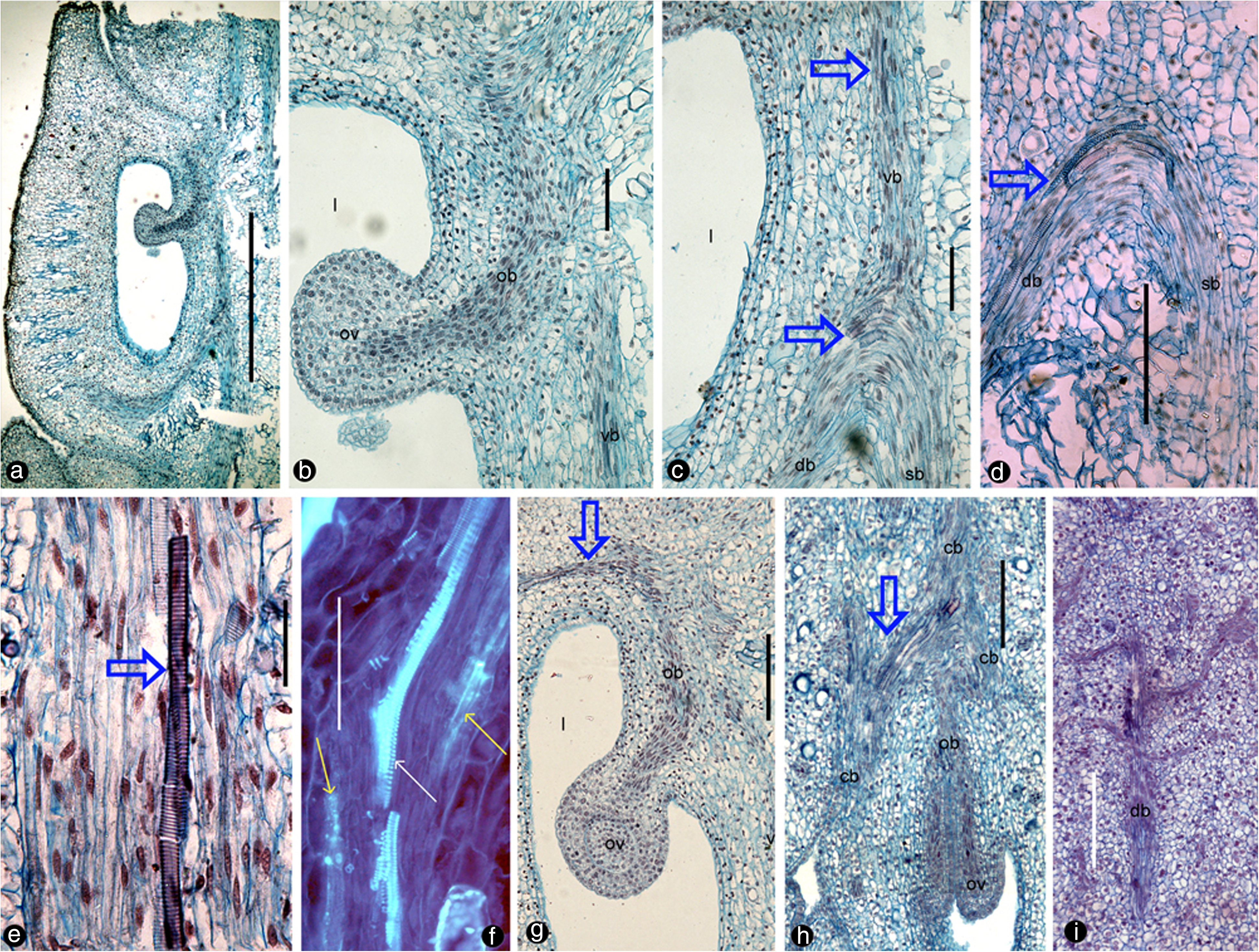

Supplement: Supplementary file 2 — Authors’ original file for figure 2 [file 40529_2014_84_MOESM2_ESM.tif]

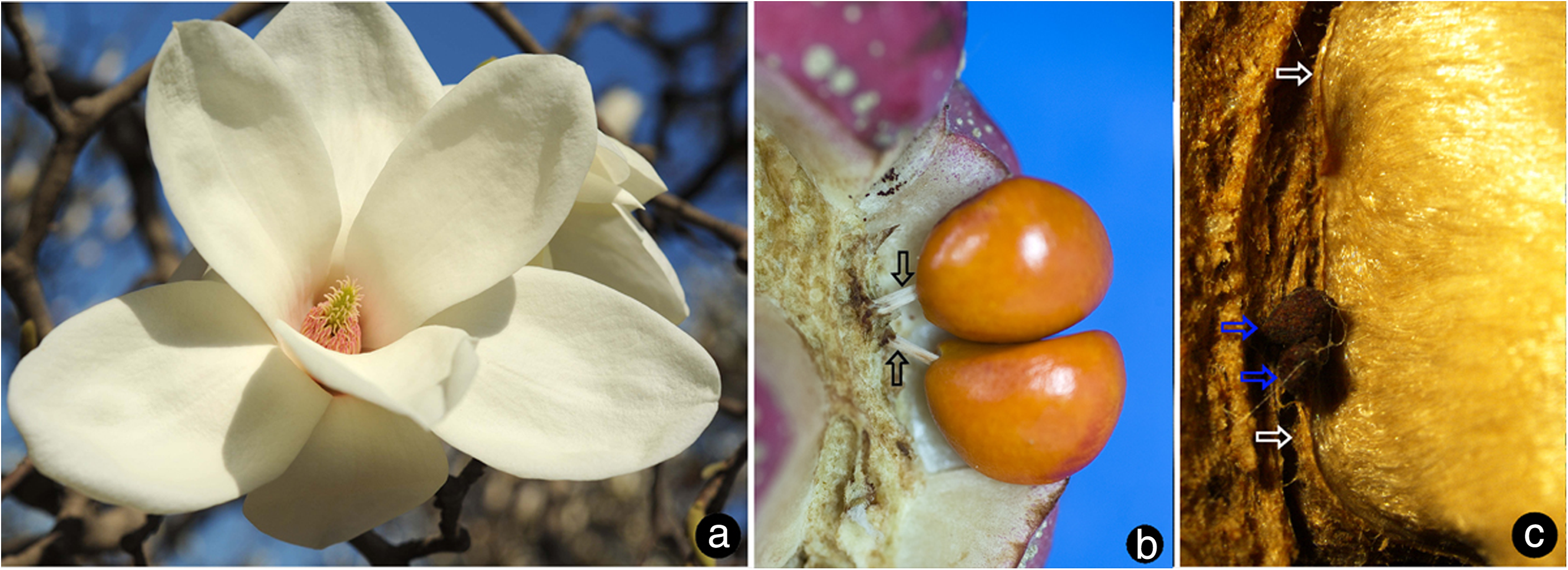

Supplement: Supplementary file 3 — Authors’ original file for figure 3 [file 40529_2014_84_MOESM3_ESM.tiff]

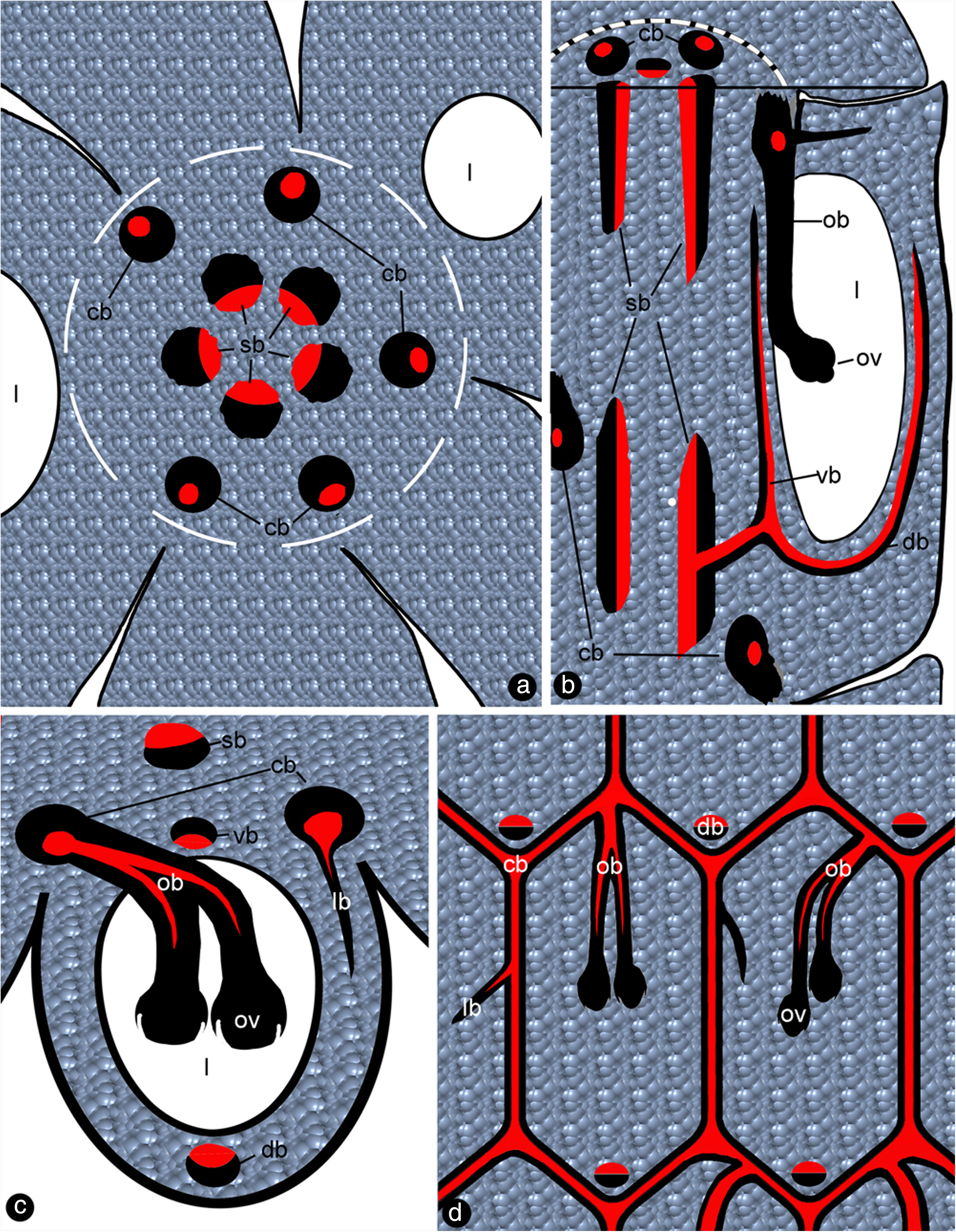

Supplement: Supplementary file 4 — Authors’ original file for figure 4 [file 40529_2014_84_MOESM4_ESM.tiff]

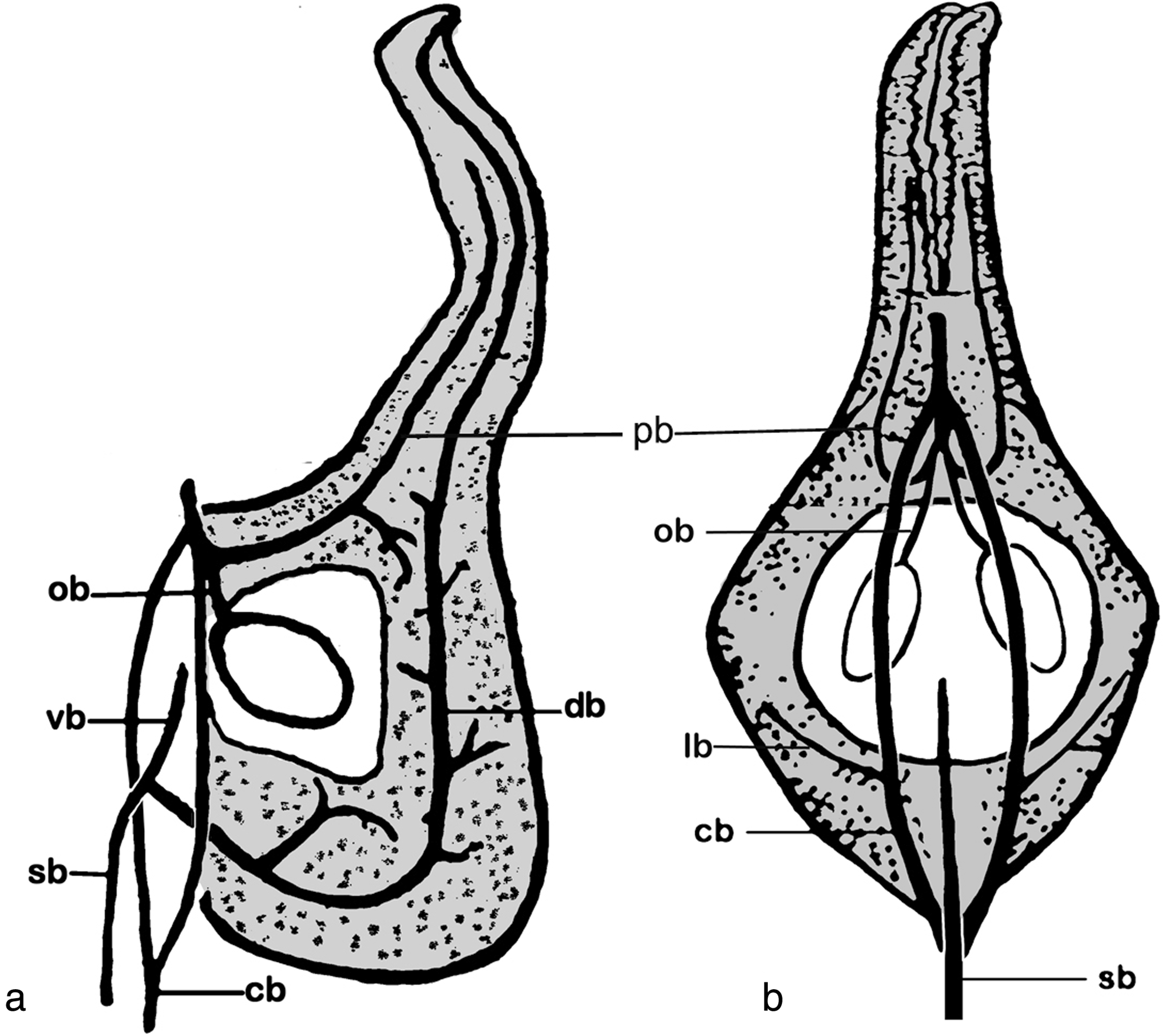

Supplement: Supplementary file 5 — Authors’ original file for figure 5 [file 40529_2014_84_MOESM5_ESM.tiff]

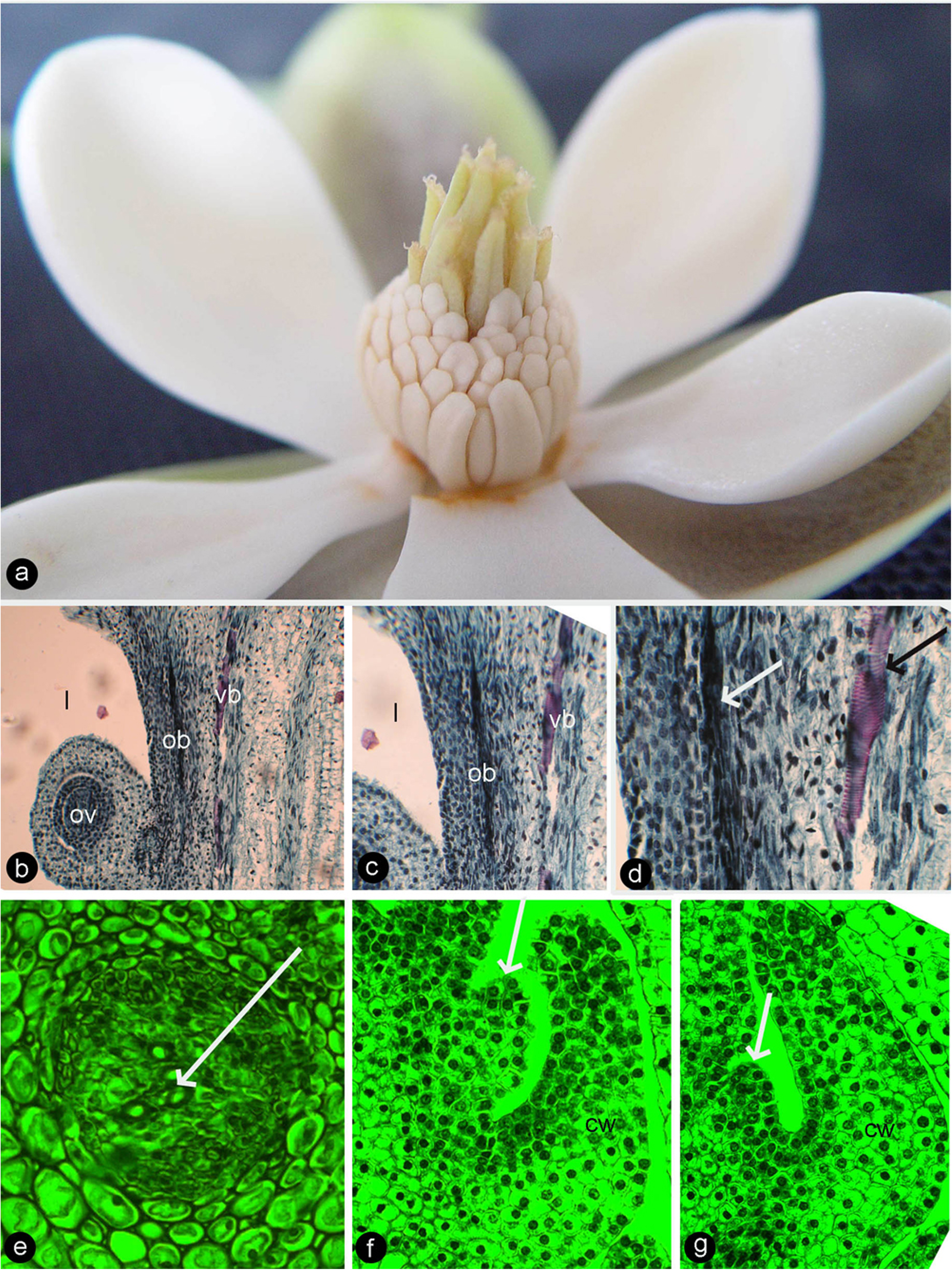

Supplement: Supplementary file 6 — Authors’ original file for figure 6 [file 40529_2014_84_MOESM6_ESM.tiff]
